# Item difficulty of eight tests for the examination of lumbar movement control in flexion in chronic non-specific lumbar back pain – a cross-sectional study

**DOI:** 10.1186/s12891-026-09798-7

**Published:** 2026-05-14

**Authors:** Anne Hoffmann, Luisa Meyering, Tom Frankenstein, Axel Schäfer, Annika Schwarz

**Affiliations:** 1 Faculty of Social Sciences, City University of Applied Sciences Bremen, Bremen, Germany; 2 Institute of Health Sciences, Department of Physiotherapy, Pain and Exercise Research Luebeck (P.E.R.L.), University of Luebeck, Luebeck, Germany; 3 Faculty of Social Work and Health, Degree Programs in Occupational Therapy, Speech Therapy, and Physiotherapy, University of Applied Sciences and Arts, Hildesheim, Germany

**Keywords:** Lumbar movement control, Non-specific chronic low back pain, Clinical tests, Inter-rater reliability, Item response theory

## Abstract

**Background:**

Non-specific chronic low back pain (NSCLBP) is a prevalent clinical condition often associated with impairments of the lumbar motor control (LMC). A test battery of 13 tests has been developed to evaluate LMC. However, the flexion-specific items demonstrated low item difficulties, limiting their diagnostic utility. Consequently, four new items for flexion were included and assessed for inter-rater reliability. The aim of this study was to evaluate and compare the item difficulty of both the original and newly proposed flexion-related LMC tests.

**Methods:**

This cross-sectional observational study included 69 participants (45 with NSCLBP, 24 without). Mean age was 50.5 years (SD 16.8) in the NSCLBP group and 45.9 years (SD 20.4) in the control group; the proportion of females was 73% (NSCLBP) and 62.5% (controls). Each participant completed eight flexion-specific LMC test items, rated as correct or incorrect by a blinded physiotherapist. In addition, participants completed questionnaires collecting demographic data and NSCLBP-specific information. Descriptive statistics were calculated, and group comparisons were performed with t-tests or Mann–Whitney U tests (significance level *p* < 0.05). Item Response Theory (IRT) analyses employing a one-parameter logistic model (1-PL) were carried out to estimate item difficulty. The test characteristic curve (TCC) and test information function (TIF) were computed for the full test battery.

**Results:**

Item difficulty of the flexion-specific items ranged from − 3.15 (easiest: forward bend) to -0.17 (most difficult: box lift). Two of the four new items exhibited higher difficulty than the existing tests. Participants with NSCLBP performed fewer flexion-specific tests correctly (mean: 5 out of 7) compared to those without NSCLBP (mean: 6 out of 7). The test battery was most informative for individuals with average to below-average LMC ability (θ range: 0 to -2.9).

**Conclusion:**

Although the newly introduced flexion items showed slightly higher difficulty, they remain insufficiently challenging to discriminate LMC deficits in individuals with mild impairments. Future research should focus on developing flexion-specific LMC tests with greater difficulty levels to improve clinical discrimination in athletic people.

**Trial registration:**

At OSF: 10.17605/OSF.IO/GR2WZ.

## Introduction

Low back pain (LBP) is one of the most prevalent clinical conditions worldwide and represents a complex epidemiological, social, and socioeconomic health challenge [1–3]. Approximately one in ten individuals with LBP develops chronic pain, necessitating long-term medical management and resulting in substantial healthcare costs [1]. Globally, LBP is the leading cause of years lived with disability, affecting 619 million people in 2020, with projections estimating an increase to 843 million by 2050 [3–5]. LBP contributes to reductions in physical and social functioning, which is associated with high long-term care expenditures [2]. This burden is expected to increase with aging populations [2]. Risk factors associated with the transition from acute to chronic LBP include female sex, unhealthy lifestyle behaviors, and psychosocial factors [6, 7].

LBP can be categorized by etiology (specific vs. non-specific), duration (acute, subacute, chronic, or recurrent), severity (assessed using subjective tools such as the Numerical Rating Scale, NRS or Visual Analogue Scale, VAS), and risk of chronicity (e.g. via the STarT Back Tool) [5]. When no specific structural or pathoanatomical cause can be identified, the condition is categorized as non-specific chronic low back pain (NSCLBP) [8].

NSCLBP is considered a multifactorial condition involving biological, psychological, and social factors and therefore requires an individualized and function-oriented therapeutic approach [9]. Several studies have reported that individuals with NSCLBP may experience an impairment in lumbar motor control (LMC) [10–12] as one of those biological factors. LMC refers to the ability to actively regulate movements of the lumbar spine during limb or trunk movements [13]. As LMC is associated with NSCLBP it should be included in physiotherapeutic diagnostics [9, 11, 14].

Multiple test batteries have been developed to assess LMC [14–17]. One of them, the TEBA-Give test battery proposed by Adelt et al. [17], is a standardized clinical test battery consisting of 13 movement control tests designed to assess direction-specific lumbar motor control in flexion, extension, and rotation/lateral flexion. The full test battery is described elsewhere [17]. These 13 physical tests are referred to as items. This directional approach is clinically meaningful and aligns with motor control concepts by Comerford & Mottram [18] and Sahrmann et al. [19].

However, studies by Adelt et al. [17] and Wend et al. [20] showed that the flexion-specific items in the TEBA-Give test battery exhibited item difficulty values that were very low. Consequently, these items may have limited ability to differentiate between individuals with and without impairments in lumbar motor control, particularly in populations with relatively good movement control. These findings highlight the need for more challenging flexion-specific tasks within the battery.

To address this limitation, Frankenstein [21] identified four additional flexion-related items — waiter’s bow, deep squat, box lift, and bilateral knee extension. In a first step the reliability of these items was examined with NSCLBP patients, and seven out of the eight test items (including the four original and four new items) demonstrated moderate to substantial inter-rater agreement, with Gwet’s AC values ranging from 0.53 to 0.76. The exception was the waiter’s bow, which showed only fair agreement (Gwet’s AC = 0.38). While these findings support the reliability of the test procedures, the psychometric properties of the new items — particularly their item difficulty — have not yet been evaluated.

Therefore, it remains unclear whether the newly proposed items are sufficiently challenging to improve the discriminatory power of the flexion component of the TEBA-Give test battery. Given the insufficient difficulty of the original flexion items and the preliminary evidence supporting the reliability of the newly proposed items, further empirical evaluation is warranted. The aim of the present study was therefore to assess and compare the item difficulty of the four original and the four newly proposed flexion-specific LMC tests by Frankenstein [21] using item response theory. The results aim to inform the selection of diagnostic tests with optimized psychometric properties, thereby improving the precision of flexion-specific LMC assessment in individuals with NSCLBP.

## Methods

This observational cross-sectional study was preregistered on the Open Science Framework (OSF.io) using a standardized template provided by AsPredicted.org (10.17605/OSF.IO/GR2WZ). Ethical approval was obtained from the institutional Ethics Commission of the University of Applied Sciences and Arts, Hildesheim (HAWK), and all participants provided written informed consent.

### Participants

The sample size for the item response theory (IRT) analysis was estimated based on recommendations for Rasch-type models regarding the precision of item parameter estimation for one parameter (1-PL IRT model). Following Linacre and Wright [22], the required sample size can be approximated using the expected standard error (SE) of item difficulty estimates and the desired confidence interval. Assuming a standard error of SE = 1 for the item difficulty parameter and a confidence interval of 99%, a minimum sample size between 27 and 61 participants is considered sufficient to obtain stable parameter estimates. To account for potential dropouts or exclusions and to increase the robustness of the estimates, a larger number of participants was recruited than the minimum required sample size. Participants were recruited between January 19, 2024, and April 25, 2024, from outpatient physiotherapy clinics, rehabilitation centers, and sports rehabilitation groups in Bremen, Hamburg, Lemgo, and Bocholt in Germany. The inclusion and exclusion criteria were aligned with previous studies by Adelt et al. [17], Wend [20], and Frankenstein [21] to ensure comparability with previous datasets. Eligible participants were adults aged 18 years or older who were able to understand instructions. Participants were included if they either had no history of NSCLBP or fulfilled the criteria for NSCLBP, defined as persistent or recurrent low back pain for at least three months, with or without radiating leg pain. Exclusion criteria included specific spinal pathologies such as vertebral fractures, inflammatory spinal diseases, spinal tumors, radiculopathy, neurological deficits, or previous spinal surgery. These criteria were based on previous studies investigating lumbar movement control in individuals with low back pain with the TEBA-Give battery and its modifications [17, 20, 21]. Potential participants were first screened by telephone for key eligibility criteria. Those who appeared eligible were invited for an on-site assessment, where inclusion and exclusion criteria were confirmed before testing.

### Procedure

**Fig. 1:**
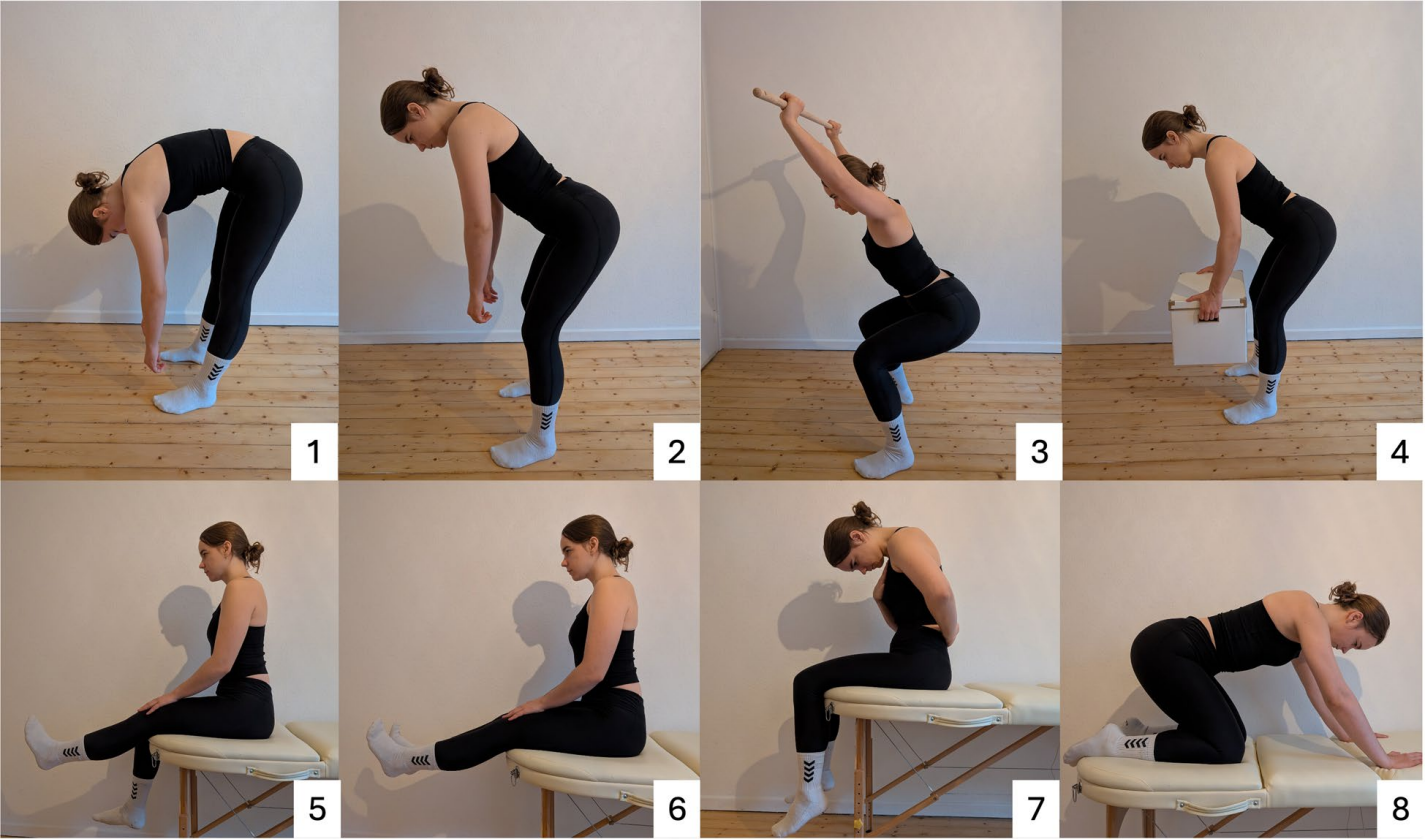
Flexion-specific lumbar movement control (LMC) test items including four original flexion-specific items from the TEBA-Give battery [17] and four newly proposed Items from Frankenstein [21], item 1 forward bend – old, item 2 waiter's bow – new, item 3 deep squat – new, Item 4 box lift – new, item 5 unilateral knee extension – old, item 6 bilateral knee extension – new, item 7 chest drop – old, item 8 rocking backwards - old

All assessments were performed using a standardized testing protocol defined prior to data collection. Participants were tested individually. The physiotherapists conducting the tests were blinded to participants’ back pain status. Each of the eight test items targeting flexion-specific LMC was explained to the participants using a standardized script. Every item was performed three times, with the starting position consistently resumed after each trial. Test execution was visually assessed in real time. The test items are illustrated in Fig. 1, and a detailed description of the item implementation is provided in the article by Frankenstein [21].

### Data collection

Data collection was conducted by two state-registered physiotherapists. Both were trained by TF in a 120-minute session to assess the four old and four new items, and they rehearsed the protocol before the pilot phase. Prior to the main study, a pilot phase was carried out to assess procedural clarity and feasibility, and to ensure consistency in test application. To maintain examiner blinding one physiotherapist was responsible for clinical testing, while the second physiotherapist — blinded to test performance — collected sociodemographic and clinical data using structured questionnaires.

Collected variables included age, sex, body weight, height, and back pain-related clinical characteristics. Participants completed validated German versions of the Fremantle Back Awareness Questionnaire (FreBAQ) [23], the Fear Avoidance Beliefs Questionnaire (FABQ) [24], and selected items from the German Pain Questionnaire (DSF) [25]. Additionally, the Oswestry Disability Index (ODI) [26] was administered, extending the questionnaire set previously used by Adelt et al. [17]. Both perceived occupational physical strain on the back and self-reported participation in sport were indicated on a rating scale of 0–10. Here, 0 defines a very low level of occupational physical strain or a very low level of self-reported participation in sport and 10 a very high level of occupational physical strain or self-reported participation in sport. The eight LMC test items — four already included in the TEBA-Give battery and four recently proposed by Frankenstein [21] – were rated as either correct or incorrect. Ratings were based on subjective visual inspection and categorized dichotomously.

### Data analysis

Anonymized data from the questionnaires and test ratings were initially entered into Microsoft Excel and then transferred to SPSS (version 28, Armonk, NY) for statistical analysis. Descriptive statistics and the associated significance tests were performed to characterize the overall sample and the subgroups with and without NSCLBP. Normal distribution was assessed using the Shapiro-Wilk test. Group comparisons were made using independent t-tests or Mann-Whitney U tests, depending on the data distribution. A p-value < 0.05 was considered statistically significant.

To evaluate the psychometric properties of the eight test items, IRT analysis was applied using STATA (version 16.1, StataCorp, College Station, TX) [27, 28]. A one-parameter logistic model (1-PL) was used, consistent with previous studies [17]. The visualizations of the results were generated in Stata (version 16.1) based on the fitted 1-PL model and exported for manuscript preparation. The 1-PL model assumes that item performance is determined solely by the latent trait — in this case, lumbar motor control in flexion (LMCFLEX). Item difficulty, test information function (TIF), item information function (IIF), and test characteristic curve (TCC) were computed. For subgroup comparison, a “multiple-group” IRT analysis was conducted. The NSCLBP group served as the reference group (θ = 0, SD = 1), and the asymptomatic control group was the focus group [29]. This analysis aimed to determine whether LMCFLEX ability differed systematically between the two populations. The statistical significance of the difference was estimated using t-test. To minimize potential bias, the examiner responsible for administering and evaluating the test items was blinded to participants’ group allocation. Pretesting of the items was implemented in the pilot phase to minimize inter-rater variability, a predefined study protocol and a standardized operating procedure were strictly followed during test instruction to ensure consistency and methodological rigor. 

## Results 

**Fig. 2:**
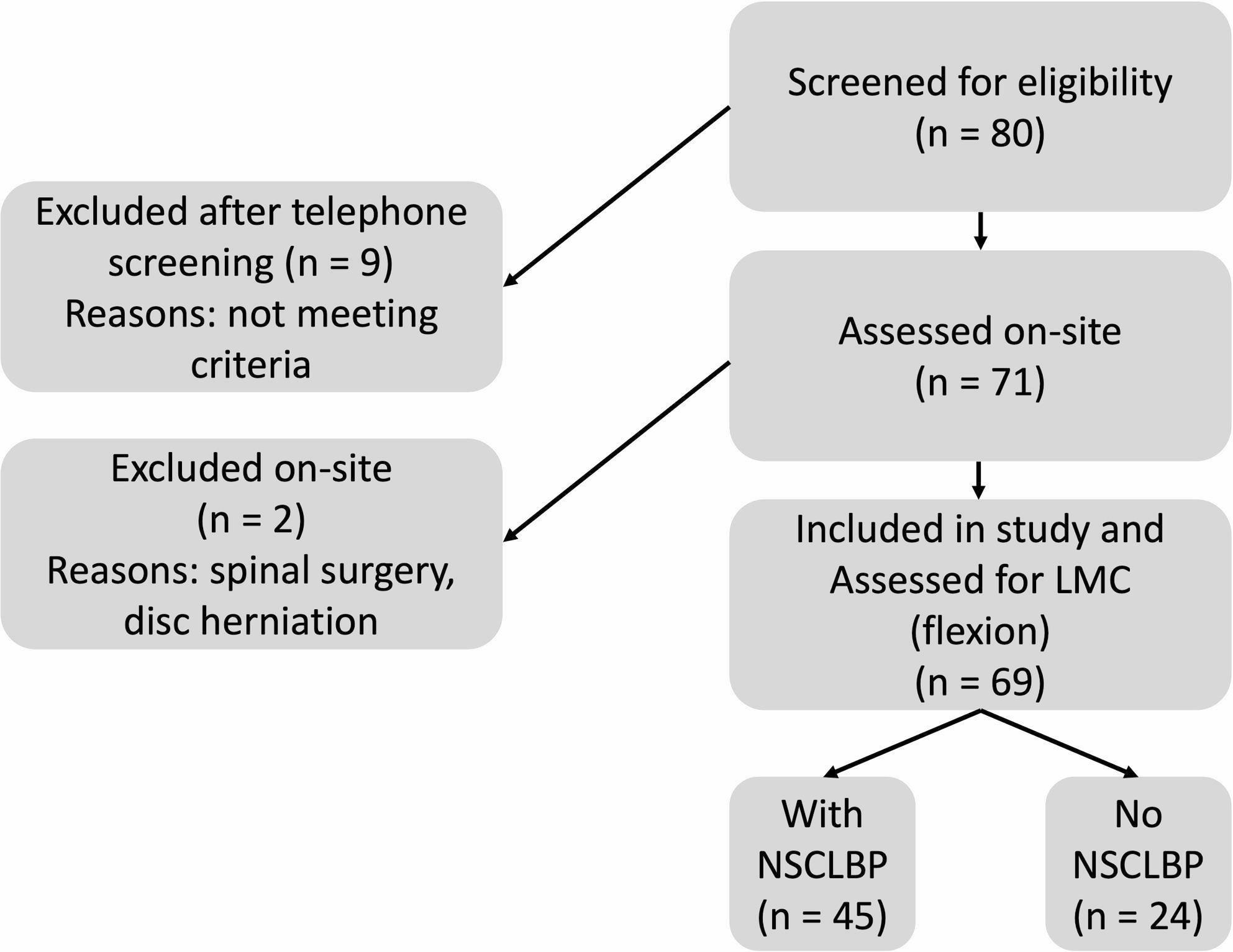
Flow of participants through the study, including assessment for eligibility, inclusion, follow-up, and analysis.

A total of 80 individuals were screened; 11 were excluded, resulting in 69 included participants (45 with NSCLBP and 24 controls) (Figure 2). Included participants were assessed for lumbar motor control (LMC) in the flexion direction. The entire sample consisted of 30 % women and had a mean age of 48.9 (SD 18.1) years. Table 1 summarizes the socio demographic characteristics of the overall sample and both groups. There were no significant group differences for age, sex, height, or weight. However, statistically significant differences were observed for occupational physical strain on the back and self-reported participation in sport. Participants with NSCLBP reported higher physical strain (median = 6, inter quartile range (IQR) = 1) than participants without NSCLBP (median = 3, IQR = 3.5; p < 0.001). Conversely, the control group reported slightly higher self-reported participation in sport (median = 7, IQR = 1) than the NSCLBP group (median = 6, IQR = 3; p = 0.012).

**Table 1:** Sociodemographic data of the participants

Variable		Group without NSCLBP (*n* = 24)	Group with NSCLBP (*n* = 45)	total (*n* = 69)	*p*-value
age in years, M (SD)		45.88 (20.35)	50.49 (16.80)	48.88 (18.09)	0.079^1^
sex, n (%)	male	9 (37.5%)	12 (27%)	21 (30%)	
	female	15 (62.5%)	33 (73%)	48 (70%)	0.352^3^
body size in cm, M (SD)		173.67 (1.25)	172.64 (1.84)	172.35 (8.13)	0.530^1^
bodyweight in kg, M (SD)		72.50 (12.84)	74.73 (13.85)	73.96 (13.28)	0.909^1^
Body-Mass-Index in kg/cm2, M (SD)		24 (3,7)	25.35 (2,1)	24.88 (4.0)	0,193^1^
occupational physical strain on the back, m (IQR)		3 (3.5)	6 (1)	6 (4)	**< 0.001**^**2**^
self-reported participation in sport, m (IQR)		7 (1)	6 (3)	6 (2)	**0.012**^**1**^

*M* Mean value, *SD* Standard deviation, *m* Median, *IQR* Interquartile range, *NSCLBP* Non-specific chronic low back pain, ^1^independent t-Test; ^2^Mann-Whitney U test; ^3^Chi square test, significant if < 0.05, significant values are marked in bold

Participants in the NSCLBP group had a median current back pain intensity of 3 (IQR = 4) out of 10. The mean values of the ODI sum score indicate mild impairment due to the NSCLBP [26] (mean = 7.76, SD = 3.94). Detailed clinical characteristics of the NSCLBP-group are presented in Table 2.

**Table 2:** NSCLBP-specific characteristics of the participants

Variable	Value	Group with NSCLBP (*n* = 45)
Current lower back pain, n (%)	Yes	30 (67%)
	No	15 (33%)
Current back pain (NRS), m (IQR)		3 (4)
Intensity of average back pain in the last 3 months, m (IQR)		4 (2.5)
FreBAQ sum score, M (SD)		7.36 (6.27)
ODI sum score, M (SD)		7.76 (3.94)
FABQw, M (SD)		11.84 (5.49)
FABQpa, M (SD)		12.6 (7.49)

*M *Mean value, *SD* Standard deviation, *m* Median, *IQR* Interquartile range, *NSCLBP* Non-specific chronic low back pain, *NRS* Numeric rating scale, *FreBAQ* Fremantle Back Awareness Questionnaire, *ODI* Oswestry Disability Index, *FABQw* Fear Avoidance Beliefs Questionnaire work subscale, *FABQpa* Fear Avoidance Beliefs Questionnaire physical activity subscale

### Scoring of flexion-specific items

Each of the 69 participants completed eight flexion-specific test items, resulting in a total of 552 individual data points. Across the entire sample, 22% of the items were performed incorrectly, while 78% were rated as correct (Table 3). Among participants without NSCLBP, 17% of the items were executed incorrectly, whereas among participants with NSCLBP, 25% of the items were performed incorrectly.

**Table 3:** Descriptive statistics of the eight flexion-specific items

Item No.	Item	Subgroup without NSCLBP (*n* = 24)		Subgroup with NSCLBP (*n* = 45)		Total group (*n* = 69)		*P*-value of the difference between the subgroups
	Evaluation	Not correct	correct	Not correct	correct	Not correct	correct	
1	Forward bend	1 (4%)	23 (96%)	3 (7%)	42 (93%)	4 (6%)	65 (94%)	0.672
2	Waiters bow	9 (38%)	15 (62%)	16 (36%)	29 (64%)	25 (36%)	44 (64%)	0.873
3	Deep squat	3 (12%)	21 (88%)	7 (16%)	38 (84%)	10 (14%)	59 (86%)	0.731
4	Box lift	9 (37%)	15 (63%)	23 (52%)	22 (48%)	32 (46%)	37 (54%)	0.280
5	Unilat knee extension	0 (0%)	24 (100%)	2 (4%)	43 (96%)	2 (3%)	67 (97%)	0.295
6	Bilat knee extension	1 (4%)	23 (96%)	5 (11%)	40 (89%)	6 (9%)	63 (91%)	0.352
7	Chest drop	3 (13%)	21 (87%)	12 (27%)	33 (73%)	15 (22%)	54 (78%)	0.174
8	Rocking backwards	6 (25%)	18 (75%)	22 (49%)	23 (51%)	28 (41%)	41 (59%)	0.054
	Full test battery	32 (17%)	160 (83%)	90 (25%)	270 (75%)	122 (22%)	430 (78%)	**0.027**

Number and percentage (%) of correctly or incorrectly completed items in the subgroups with and without Non-specific Chronic Low Back Pain (NSCLBP) and in the overall group; *p* = chi-square test, significant if < 0.05, significant values are marked in bold

Table 3 displays item-by-item performance across both subgroups. Item 1 (forward bend) showed the highest rate of correct performance (94%), whereas item 4 (box lift) showed the lowest (54%). The difference in total correct performance between groups was statistically significant (*p* = 0.027), with the NSCLBP group correctly performing on average 5 out of 7 items (excluding item 5), and the control group 6 out of 7.

Due to a very high correlation of *r* = 1 between item 5 (unilateral knee extension) and item 6 (bilateral knee extension), with all incorrect responses on item 5 mirrored in item 6, item 5 was excluded from the IRT analysis to prevent model distortion.

### Item response theory analysis

**Fig. 3:**
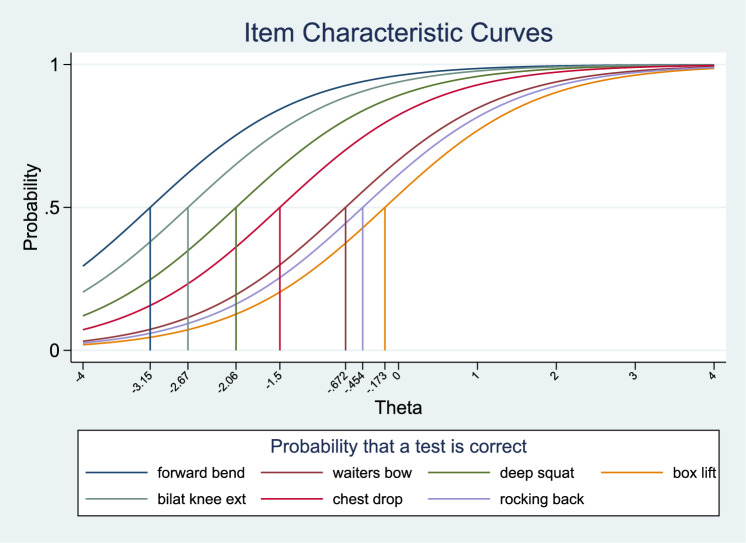
Item Characteristic Curve (ICC) graph of the item characteristic curve for seven items with added difficulty values: Y-axis: probability of a positive test result. X-axis: Theta values; Theta, indicating the degree of ability to control the movements of the lumbar spine in flexion (higher values = better performance). The values given indicates the midpoint probability. The colors indicate the different items. Figure generated in Stata (version 16.1).

Item parameters for the full sample are presented in Fig. 3. The difficulty parameter indicates the relative difficulty of each item, with the easiest item having the lowest value. Item difficulty values ranged from − 3.15 (forward bend, easiest) to − 0.17 (box lift, most difficult), based on the entire sample (participants with and without NSCLBP). The item characteristic curves (ICCs) illustrate the probability of correct performance across LMC_FLEX_ ability levels (theta values, Fig. 3). For example, a difficulty value of − 3.15 indicates that a participant with a theta score 3.15 below the sample mean has a 50% chance of correctly performing the forward bend.

**Fig. 4:**
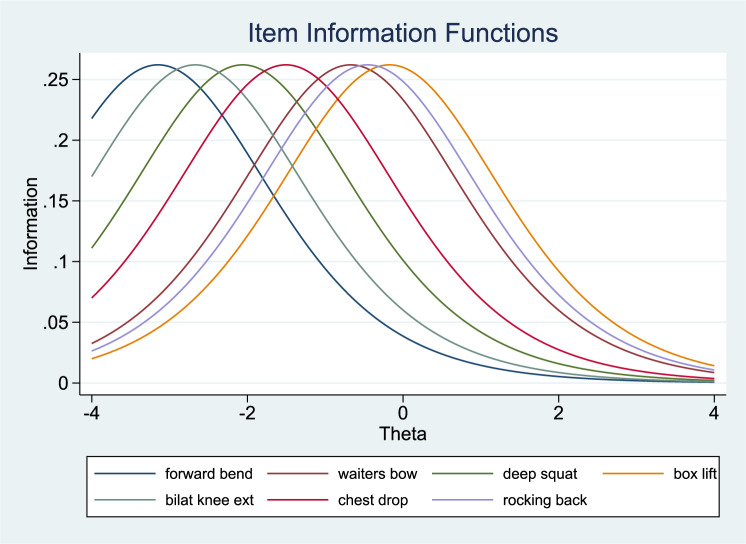
Item Information Function for the seven items. Y-axis: amount of information. X-axis: Theta values; Theta, indicating the degree of ability to control the movements of the lumbar spine in flexion (higher values = better performance). Figure generated in Stata (version 16.1)

**Fig. 5:**
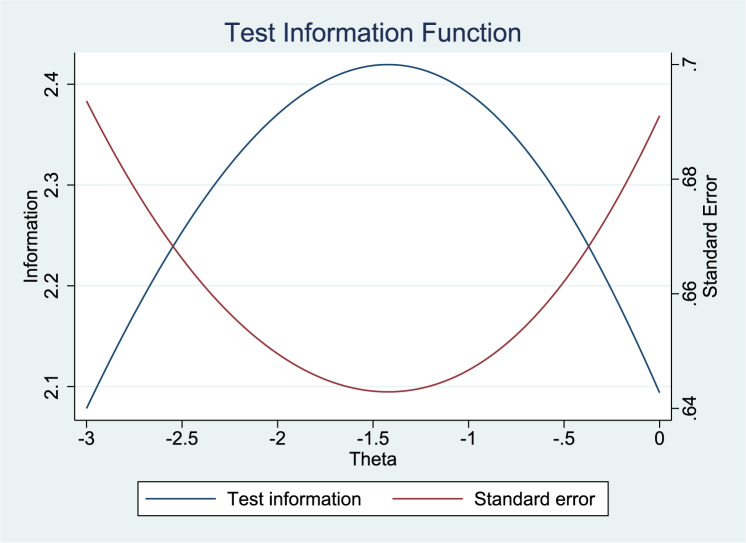
Test Information Function of the whole test battery. X-axis: theta values left y-axis (blue line) amount of test information right y-axis (red line) Standard error. It can be seen that the standard error decreases when the test information increases. Theta, indicating the degree of ability to control the movements of the lumbar spine in flexion (higher values = better performance). Figure generated in Stata (version 16.1)

The item information function (IIF, Fig. 4 ) and test information function (TIF, Fig. 5) showed that, the items related to flexion were the most informative for participants, with a theta range between 0 and − 2.9. Within this range, items provided the highest information (> 2.1) and the lowest measurement error (standard error < 0.69). Item 1 (forward bend) was most informative for participants with below-average LMC_FLEX_ ability, while item 4 (box lift) provided the greatest differentiation around the average ability level.

### Group comparison using IRT

**Fig. 6:**
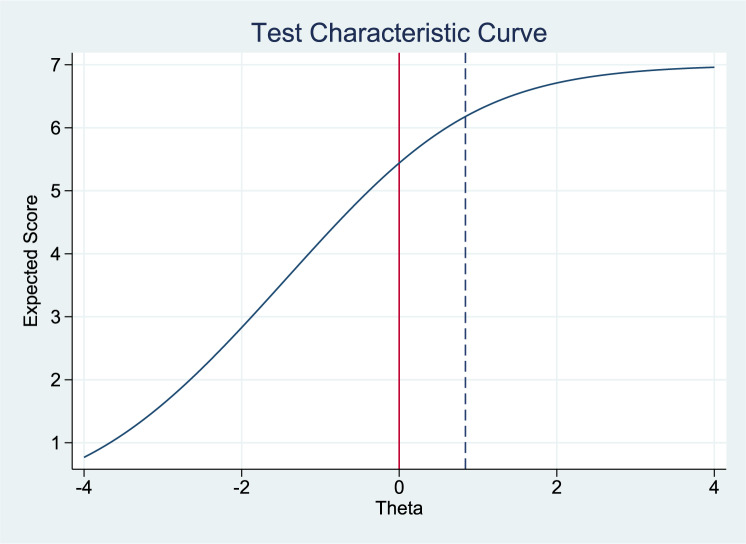
Test characteristic curve comparing both groups. Red line mean value of group without NSCLBP, blue dashed line mean value of group with NSCLBP. Y-axis: classical sum score of correct performed number of tests. X-axis: Theta values. Figure generated in Stata (version 16.1)

The multiple-group IRT analysis revealed that individuals without NSCLBP had significantly better flexion-related LMC ability (difference = 0.84, p =. 0001). The items can be utilized equally well across both subgroups (with and without NSCLBP). Using the NSCLBP group as the reference (mean theta = 0, SD = 1), the group without NSCLBP demonstrated a mean theta of 0.84, indicating a higher average LMC_FLEX_ ability. Based on the test characteristic curve (TCC, Fig. 6), participants with NSCLBP were expected to perform approximately 5 out of 7 items correctly, whereas asymptomatic participants were expected to correctly perform 6 out of 7.

## Discussion

This study aimed to evaluate and compare the item difficulty of four original and four newly proposed test items assessing LMC in the flexion direction using IRT. The results indicate that while some newly proposed tasks were more difficult than the original ones, all flexion items still showed negative difficulty parameters. This suggests that, relative to the ability distribution of the present sample, the flexion items are generally easy and mainly targeted at individuals with average to below-average LMC_FLEX_ ability. Consequently, the flexion item set may have limited discriminatory capacity for detecting subtle deficits in higher-functioning individuals.

### Interpretation of item difficulty

The comparison between original and newly proposed flexion items revealed that the new tasks — particularly box lift and waiter’s bow — were generally more difficult than the original items. Item 1 (forward bend) was identified as the easiest (difficulty = − 3.15), and item 4 (box lift) as the most difficult (difficulty = − 0.17). In the IRT, the item difficulty parameter represents the ability level (θ) at which the probability of correct performance is 50% [30]. Thus, negative difficulty values indicate that at the reference mean ability (θ = 0) the probability of correct performance exceeds 50% for all items. Accordingly, the flexion items are primarily informative in the lower ability range and provide limited measurement precision in the higher-ability range. This is consistent with the test information results, showing the highest information for θ values approximately between 0 and − 2.9.

These findings extend previous IRT-based work on direction-specific LMC testing, which suggested that flexion items tend to exhibit comparatively low difficulty and may therefore be prone to ceiling effects, whereas other movement directions may cover higher difficulty ranges [17]. One possible explanation for the generally low item difficulty observed in the present study may be that flexion-related motor control tasks place relatively low mechanical demands on lumbar stability compared with other movement directions such as extension or rotation [31]. Previous research suggests that motor control deficits in individuals with NSCLBP may be more pronounced in tasks requiring higher spinal stability or load transfer [14, 17]. This may partly explain why flexion-specific tests show limited discriminatory capacity, particularly in individuals with relatively good motor control.

### Group differences and the role of LMC in NSCLBP

The findings also support known associations between impaired LMC and NSCLBP. Participants with NSCLBP performed significantly fewer tests correctly than those in the control group, with an average of 5 vs. 6 out of 7 items completed correctly. These results align with previous studies [11, 14], which reported decreased LMC ability in people with NSCLBP. Compared to Wend et al. [20], who reported smaller performance differences between groups, the present study showed clearer group separation — potentially related to the higher number of evaluated flexion items and the inclusion of comparatively more difficult tasks. This suggests a somewhat stronger differentiation between the subgroups regarding LMC in this study compared to Wend et al. [20]. Since individuals with LBP tend to have poorer LMC on average [11, 14] the increased item difficulty helps to better reveal the differences between groups.

At the same time, clinically assessed lumbopelvic sensorimotor/movement control tests have been discussed critically regarding their overall validity. A recent COSMIN-based systematic review concluded that the quality of evidence for convergent and known-groups validity of clinically assessed lumbopelvic sensorimotor control tests is (very) low, and that test outcomes should be interpreted cautiously when used for clinical decision-making [32]. The present results should therefore be interpreted as a psychometric contribution to refining the flexion component (targeting and item difficulty) rather than as proof of standalone diagnostic validity.

### Implications for the TEBA-give test battery

Given that all eight flexion items showed low item difficulty, it may be reasonable to selectively incorporate only the most challenging flexion items into the TEBA-Give test battery. This might improve targeting of the flexion subscale and to reduce ceiling effects and increase the diagnostic sensitivity of the battery. Based on the results, the box lift and waiter’s bow from the new items, and rocking backwards and chest drop from the original set, exhibited the highest difficulty values. Conversely, forward bend — the easiest item — may not contribute meaningful information and could be considered optional depending on the intended purpose (screening vs. assessment across a broader ability range). Incorporating these findings would result in a revised test battery still comprising 4 flexion-specific items and 13 items in total. This recommendation aligns with findings by Adelt et al. [17], who also identified Rocking Backwards as the most difficult among the original four flexion items.

### Clinical implications

In clinical practice, direction-specific LMC testing is valuable for hypothesis generation and tailoring therapy to individual motor control deficits, particularly when combined with patient history and other examination findings. The results of the item difficulty for both the existing and new flexion items all display negative values across the overall sample, indicating that more than 50% of participants correctly perform these items. This suggests that the items are primarily informative for individuals with lower LMC_FLEX_ ability and may show ceiling effects in higher-functioning individuals. In patients with NSCLBP, the low item difficulty reduces the ability to distinguish between adequate and limited LMC. Adelt et al. [17] recommend that clinicians use the most challenging item in each movement direction as a screening tool. Based on the current findings, box lift appears to be the most appropriate flexion item for this purpose. When the aim is to identify more subtle impairments, clinicians may consider using the most challenging flexion tasks or complementing flexion testing with items from other movement directions. Knowledge of item difficulty can also support a more time-efficient, stepwise assessment and targeted exercise selection. Clinicians may start with an item matching the suspected impairment level and progress only if needed; easier items can be used as home exercises once performed correctly, while more difficult items may be practiced under supervision. Thus, IRT-derived difficulty estimates provide an empirical rationale for clinical decision-making that is otherwise largely based on experience.

### Future research

Future studies should aim to design and validate flexion-specific tasks that better target the mid- to high-range of the LMC_FLEX_ spectrum, thereby enhancing discriminatory power. Given the task-dependent nature of motor control and movement quality and the absence of a single criterion standard, future work should also place greater emphasis on content validity by mapping test tasks to the specific motor control functions they are intended to assess [33]. In addition, it remains unclear whether deficits in flexion control are generally less pronounced in NSCLBP than in other movement directions (e.g., extension or rotation). This should be examined in comparative studies that analyze all directional subcomponents of LMC. Lastly, the influence of patient characteristics (e.g., age, pain duration, disability level) on item performance warrants further investigation.

### Strengths and limitations

This study has several methodological strengths, including blinded test procedures, standardized protocols, and validated questionnaires (ODI, FABQ, FerBaQ). Measuring pain-related disability using the ODI enhanced the comparability of the present findings with previous studies investigating lumbar motor control in individuals with chronic low back pain. Several earlier studies assessing motor control deficits have reported associations between disability levels and motor control performance. Including the ODI therefore allowed a more comprehensive characterization in comparison to existing studies in this field, such as that of Luomajoki et al. [34]. The test instruction for the Chest Drop was modified in the present study, as Frankenstein [21] observed frequent incorrect execution of the original version. To address this, greater emphasis was placed on initiating movement from the upper spine in the direction of flexion, which may limit comparability with previous studies employing the original instruction. A pilot phase was conducted to reduce inter-rater variability and ensure procedural reliability.

However, several limitations must be acknowledged. First, unilateral knee extension was excluded from the IRT analysis due to redundancy with bilateral knee extension. This may have affected the psychometric balance of the item set. Second, each participant completed 24 test repetitions (3 trials × 8 items), which may have led to fatigue effects and potential underperformance. Furthermore, as all items were presented in a fixed order across participants, order effects cannot be ruled out. Finally, it should be noted that the items examined in this study assed LMC exclusively in the flexion direction. As a result, a comprehensive evaluation of overall LMC requires inclusion of the remaining items from the TEBA-Give test battery, which cover extension and rotation/lateral flexion movements.

## Conclusion

This study evaluated the item difficulty of eight flexion-specific test items used to assess lumbar motor control in individuals with and without non-specific chronic low back pain. Although some of the newly proposed items showed slightly higher difficulty than the original tests, all items demonstrated relatively low difficulty values, indicating limited ability to detect subtle motor control deficits.

These findings suggest that the flexion component of the TEBA-Give test battery may benefit from the inclusion of more challenging tasks to improve its discriminatory capacity. Future research should therefore focus on developing and validating flexion-specific tests with higher difficulty levels to better differentiate lumbar motor control impairments across different functional levels.

## Data Availability

The datasets analyzed during the current study is available from the corresponding author on reasonable request.

## Abbreviations

DSF: German pain Questionnaire
FABQ: Fear Avoidance Beliefs Questionnaire
FABQw: Fear Avoidance Beliefs Questionnaire work subscale
FABQpa: Fear Avoidance Beliefs Questionnaire physical activity subscale
FreBAQ: Fremantle Back Awareness Questionnaire
HAWK: university of applied sciences and arts
ICC: Item characteristic curve
IIF: Item information function
IQ: Interquartile range
IRT: Item response theory
CI: Confidence interval
LBP: Low back pain
LMC: Lumbar motor control
MC: Motor control
M: Mean
NRS: Numeric Rating Scale
NSCLBP: Non-specific chronic low back pain
ODI: Oswestry Disability Index
SD: Standard deviation
SE: Standard error of measurement
TIF: Test information function
TCC: Test characteristic curve
1-PL: One parameter logistic model
